# COVID-19 pandemic and mortality in nursing homes across USA and Europe up to October 2021

**DOI:** 10.1007/s41999-022-00637-1

**Published:** 2022-03-17

**Authors:** Ulla L. Aalto, Kaisu H. Pitkälä, Karen Andersen-Ranberg, Sylvie Bonin-Guillaume, Alfonso Jose Cruz-Jentoft, Maria Eriksdotter, Adam L. Gordon, Markus Gosch, Iva Holmerova, Hannu Kautiainen, Miia Kivipelto, Jurate Macijauskiene, Desmond O’Neill, Nele van den Noortgate, Anette H. Ranhoff, Jos M. G. A. Schols, Katrin Singler, Mindaugas Stankunas, Joseph G. Ouslander

**Affiliations:** 1 Department of Social Services and Health Care, Home-Care Services, City of Helsinki, Finland; 2 Department of General Practice and Primary Health Care, University of Helsinki, Helsinki, Finland; 3 Unit of Primary Health Care, Helsinki University Hospital, Helsinki, Finland; 4 Department of Geriatric Medicine, Odense University Hospital, Odense, Denmark; 5 Geriatric Research Unit, Dept of Clinical Research, University of Southern Denmark, Odense, Denmark; 6 Department of Internal and Geriatric Medicine, University Hospital of Marseille, Aix Marseille University, Marseille, France; 7 Servicio de Geriatría, Hospital Universitario Ramón y Cajal (IRYCIS), Madrid, Spain; 8 Division of Clinical Geriatrics, Department of Neurobiology, Care Sciences and Society, Karolinska Institutet, Stockholm, Sweden; 9 Theme Inflammation and Aging, Karolinska University Hospital, Huddinge, Sweden; 10 Unit of Injury, Inflammation and Recovery Sciences, School of Medicine, University of Nottingham, Nottingham, UK; 11 Department of Geriatrics, Paracelsus Medical University Nürnberg, General Hospital Nürnberg, Nurnberg, Germany; 12 Department of Longevity Studies, Faculty of Humanities, Charles University, Prague, Czech Republic; 13 Institute of Public Health and Clinical Nutrition, University of Eastern Finland, Kuopio, Finland; 14 Department of Geriatrics, Lithuanian University of Health Sciences, Kaunas, Lithuania; 15 Department of Medical Gerontology, Trinity College Dublin, Dublin, Ireland; 16 Department of Geriatric Medicine, Ghent University Hospital, Ghent, Belgium; 17 Department of Clinical Science, University of Bergen, Bergen, Norway; 18 Norwegian Institute of Public Health, Oslo, Norway; 19 Department of Health Services Research, CAPHRI, Maastricht University, Maastricht, The Netherlands; 20 Department of Family Medicine, CAPHRI, Maastricht University, Maastricht, The Netherlands; 21 Institute for Biomedicine of Ageing, Friedrich-Alexander-University, Erlangen-Nürnberg, Germany; 22 Department of Health Management, Lithuanian University of Health Sciences, Kaunas, Lithuania; 23 Charles E. Schmidt College of Medicine, Florida Atlantic University, Boca Raton, FL USA; 24 Department of Geriatrics, Helsinki University Hospital, Helsinki, Finland

**Keywords:** Nursing home, Mortality, COVID-19

## Abstract

**Purpose:**

We compared the prevalence of COVID-19 and related mortality in nursing homes (NHs) in 14 countries until October 2021. We explored the relationship between COVID-19 mortality in NHs with the average size of NHs and with the COVID-19 deaths at a population level.

**Methods:**

The total number of COVID-19 cases and COVID-19-related deaths in all NHs as well as the total number of NHs and NH beds were provided by representatives of 14 countries. The population level respective figures in each country were provided up to October 2021.

**Results:**

There was a wide variation in prevalence of COVID-19 cases and deaths between countries. We observed a significant correlation between COVID-19 deaths in NHs and that of the total population and between the mean size of NHs and COVID-19 deaths.

**Conclusion:**

Side-by-side comparisons between countries allow international sharing of good practice to better enable future pandemic preparedness.

## Introduction

Nursing homes (NHs) worldwide have extensively suffered from the COVID-19 pandemic. The first wave during spring 2020 hit NHs especially hard since 26–80% of all worldwide COVID-19 deaths occurred in NHs [1, 2]. This was due to several reasons, such as insufficient knowledge of the disease, lack of personal protective equipment, and shortage of diagnostic capacity. Most NH residents suffer from multimorbidities and considerable handicaps, and therefore, often show impaired immunity and malnutrition. Thus, NH residents living with frailty and disability have been extremely vulnerable to the virus. Furthermore, NHs provide “high-touch care”. This indicates that most NH residents need professionals’ assistance in dressing, bathing, toileting and/or in mobility. They share bathrooms, dining rooms and group activities. In addition, prevalent cognitive impairment means residents are less able to comply with quarantine rules and may walk around NHs, including into other residents’ rooms.

Until February 2021, a mean 41% of all worldwide COVID-19 deaths took place in long-term care [3, 4]. However, there are marked differences in the spread and mortality figures between countries. The factors behind the variations between NHs include resident-related factors, facility and staffing characteristics [3, 5, 6]. It has been suggested that high-quality NHs have lower prevalence of COVID-19 cases than those with lower quality although confounding factors may underlie this [7]. However, the full range of factors behind the wide variations between countries remains unclear. Most studies published so far report only the mortality rates during the first wave of COVID-19 (March to May 2020). To our knowledge, the latest figures reported are from February 2021 [3, 4].

The aim of this study was to compare prevalence of COVID-19 cases and related mortality in NHs in 14 countries up to October 2021. We explored the relationship between COVID-19 mortality in NHs with the average size of NHs in each country and with the COVID-19 deaths at a whole population level.

## Methods

The figures were provided by representatives of the US and 13 European countries and include total COVID-19 cases and COVID-19-related deaths in all NHs as well as respective figures at the whole population level in each country from the beginning of the pandemic up to October 2021. Contributors from each country also provided the total number of NHs and NH beds in their country. The population data and figures for the total number of cases and deaths in each country were verified from www.worldometers.info [8]. We also checked that our data were in line with that in the OECD (Organisation for Economic Co-operation and Development) report [3]. The data are presented as numbers and percentages. The relationship between NH deaths and population level deaths or average NH size is illustrated with figures and Pearson correlation coefficients.

## Results

**Fig. 1:**
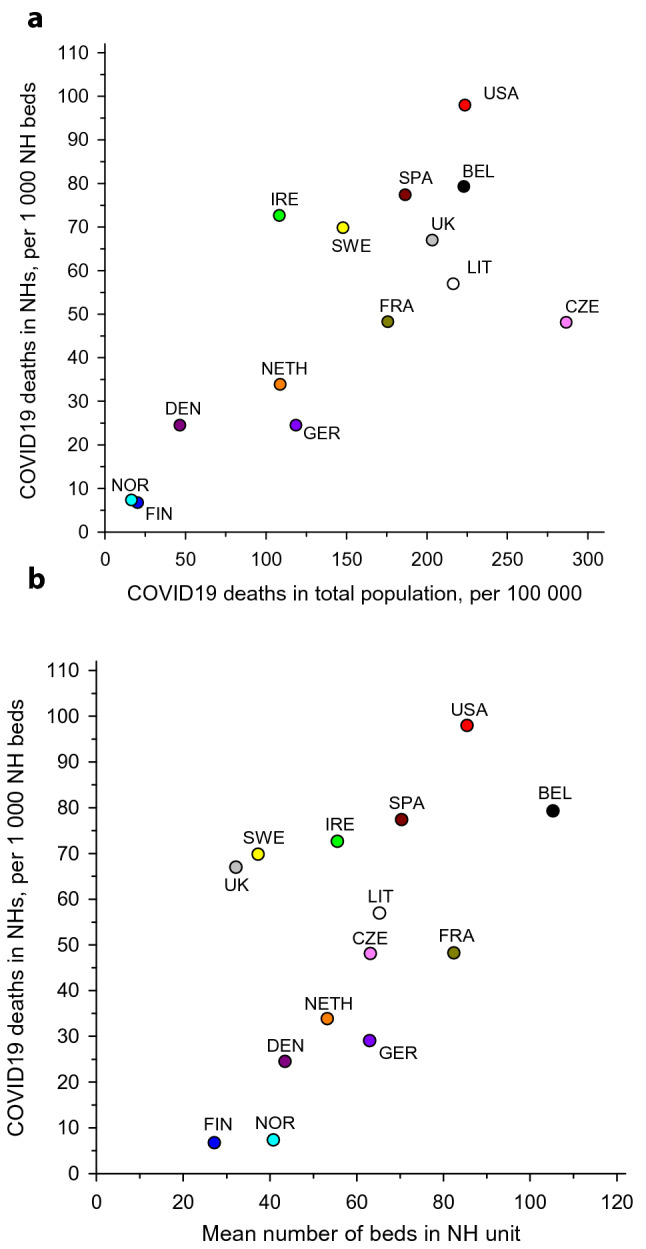
Panel **A**: relationship of COVID-19 deaths in the total population with COVID-19 deaths in nursing homes (see Table 1 for country abbreviations). Panel **B**: relationship of mean size of nursing homes (mean number of beds) with COVID-19 deaths in nursing homes (see Table 1 for country abbreviations)

The number of COVID-19 NH deaths per total population COVID-19 deaths ranged from 11% (Czech Republic) to 50% (Belgium). The proportion of COVID-19 cases per occupied NH bed ranged from 2.2% (Finland) up to 50% (USA). Some countries were unable to present these data due to lack of accurate diagnostic data especially during the first wave. The proportion of NH residents deceased due to COVID-19 per occupied NH beds ranged from 0.7% (Finland and Norway) to 9.8% (USA) (Table 1). A correlation was observed between COVID-19 deaths in NHs and deaths in the total population (*r* = 0.73). In addition, the mean size of NHs in each country and COVID-19 deaths correlated with each other (*r* = 0.56) (Fig. 1).

**Table 1:** Number of COVID-19 cases and deaths in NHs and in total population until October 2021

Country	Number of NHs (N)	Number of NH beds (N)	Average size of NH, mean number of beds (N)	Number of COVID-19 cases in NHs (% of occupied beds)	Number of COVID-19 deaths in NHs (% of occupied beds)	Total population in the country (N)	Number of COVID-19 cases in total population	Number of COVID-19 deaths in total population	Proportion of COVID-19 NH deaths/population COVID-19 deaths (%)
USA	16,381	1,400,000	85	702,285 (50)	137,126 (9.8)	333,750,000	45,900,000	746,509	18
Belgium (BEL)	1545	162,700	105	N.A	12,899 (7.9)	11,660,000	1,360,650	25,994	50
Czech Republic (CZE)	1126	71,094	63	22,696 (32)	3418 (4.8)	10,740,000	1,800,000	30,775	11
Denmark (DEN)	932	40,500	43	4072 (10)	991 (2.4)	5,820,000	381,000	2708	37
Finland (FIN)	1773	48,166	27	1066 (2.2)	323 (0.7)	5,550,000	151,787	1120	29
France (FRA)	7547	622,000	82	224,000 (36)	30,000 (4.8)	65,480,000	6,900,000	115,000	26
Germany (GER)	15,380	969,553	63	118,890 (12)	23,750 (2.4)	84,160,000	5,497,795	99,768	24
Ireland (IRE)	576	32,000	56	N.A	2324 (7.3)	5,020,000	414,802	5436	43
Lithuania (LIT)	218	14,225	65	6546 (46)	810 (5.7)	2,670,000	399,532	5776	14
The Netherlands (NETH)	2350	125,000	53	36,550 (29)	4,231 (3.4)	17,190,000	2,094,470	18,698	23
Norway (NOR)	956	39,000	41	900 (2.3)	285 (0.7)	5,480,000	200,855	900	32
Spain (SPA)	5542	390,000	70	95,291 (24)	30,170 (7.7)	46,780,000	5,004,143	87,238	35
Sweden (SWE)	2200	82,000	37	17,390 (21)	5725 (7.0)	10,190,000	1,179,192	15,065	38
UK	14,000	450,000	32	N.A	30,130 (6.7)	68,390,000	8,600,000	139,000	22

*NH* nursing home; *N.A.* not available; Abbreviations for countries correspond to Fig. 1

## Discussion

The COVID-19 mortality rate in NHs was related with COVID-19 mortality in the total population. This indicates that the spread of virus to NHs derives from the total burden of COVID-19 in the surrounding society. Second, larger average size of NHs was associated with increased mortality.

Our study findings are in line with previous data suggesting that the prevalence of COVID-19 in the community is a strong predictor of COVID-19 spread in NHs [3, 4, 9]. In addition, larger NH facilities have been associated with a greater likelihood of COVID-19 outbreaks than smaller units [5, 9]. It has become evident that the structure in many NHs is suboptimal, as several residents often share a room facilitating the spread of health care-associated infections and hampering the isolation and quarantine measures [10]. Higher occupancy rates have been shown to be associated with increased risk of COVID-19 infection [6].

As a strength, our figures cover the situation in 14 countries until October 2021, and as far as we know, there is no such coverage published earlier. Our data provide figures at each country and population level. As limitations, we do not have data on facility level resident characteristics, staffing resources or qualifications, vaccinations, resident density, or their transit to/from hospitals. These have been associated with COVID-19 infection rate and mortality [3, 5, 9, 11–13]. It is well known that the education and qualification of staffing varies greatly between the countries. The deputies working in multiple wards or facilities easily transfer virus from one NH to another [5]. Countries also vary in applying safety measures and how they allow visitors [3, 5]. Resident admissions to hospital and back have a potential for transmitting the virus.

Our findings should be interpreted with caution. There are challenges in our international comparison due to low testing capacity during the first wave and routine testing being uncommon in many countries [3]. Some countries report deaths of NH residents irrespective of their site of death, whereas others report only deaths in NHs (e.g., Finland). Some countries report only those tested positive, whereas some report also suspected cases [3]. Moreover, NHs have varying definitions depending on country [15]. In some countries such as the USA and the Netherlands, NHs provide also subacute care and rehabilitation for short-term patients which may expose residents to infections. A previous OECD report differed from our analysis by including home care clients as long-term care residents [3]. Thus, there are great challenges in exploring the reasons behind the differences in prevalence and deaths of COVID-19 among NH residents between countries. However, our study suggests that the size of NH and the burden of virus in the surrounding population had a significant role in the number of COVID-19 cases and deaths in NHs.

Nevertheless, according to previous research and our current study, some factors behind the results could be identified and, furthermore, considered in aiming at minimizing the impact of future outbreaks in NHs. Previous studies suggest that the quality of NHs defined by the staffing ratio is of great importance as regards the pandemic, since lower staffing ratio has been associated with higher COVID-19 infection rates [7, 11]. Thus, smaller NH units combined with higher staffing ratio could be a solution to prevent contagious infections. High surrounding incidence of COVID-19 should be recognized as a red flag in itself, leading to certain protective measures in NHs, such as intensified infection control.

Finally, prevalence figures change quickly and this report reflects the situation in October 2021. Today, the figures have already changed as we are faced with the Omicron wave. However, we need to illustrate how COVID-19 has affected NHs during the course of the whole pandemic since NH residents form an especially vulnerable population. The NH outbreaks highlight the need for careful review of how we provide care and pandemic resilience in NHs. These side-by-side comparisons between countries could enable international sharing of good practice to better enable future pandemic preparedness [14].
